# Probabilistic analysis of a concrete column in an aggressive soil environment

**DOI:** 10.1371/journal.pone.0212902

**Published:** 2019-03-07

**Authors:** Janusz Kozubal, Marek Wyjadłowski, Dmitri Steshenko

**Affiliations:** 1 Wroclaw University of Science and Technology, Wrocław, Poland; 2 North Caucausus Federal University, Stavropol, Russian Federation; Technische Universiteit Delft, NETHERLANDS

## Abstract

Sulphate attack is one of the most important factors that limit the lifetime of pure concrete constructions. Harsh environmental conditions have a large impact on the operational costs of concrete columns or piles dipped into soil. The results are non-deterministic; therefore, reliability analysis is often used. The strength characteristics of the substrate around the construction were modelled as one-dimensional prismatic beams related with random *p-y* curves. Sulphate deterioration is defined as a set of random variables jointed with two dimensional mechanical systems at acceptable levels. Fick’s second law describes the penetration of sulphate ingress into pure concrete with explicit numerical solutions for boundary conditions and an increase in the transition factor under the progress of sulphate ingress. This process was partially solved via analytical methods for sulphate ion transport and numerically for a random field. This solves the mechanical task and determines the system reliability. A numerical example is provided to illustrate the proposed method to prevent unexpected structural failures during column service life. The proposed methodology can assist designers and can help to make decisions on existing foundations to ensure the safety of geotechnical construction.

## 1. Introduction

During the analysis of chemical corrosion problems in concrete Controlled Modulus Columns (CMC) and similar variants of concrete piles, it is important to present the changes depending on the load carrying capacity over time. However, CMCs are often used to improve soil characteristics as a compressible soil layer in a global scale. CMCs are frequently used to reduce shear forces generated by earth pressure, slope of embankment, slipped inclined layers, and structural support elements. Other typical horizontal loads are inducted by wind and breaking forces of the pavement layer. These are carried through the soil transmission layer above the head of the columns. The approach used here is dedicated to situations where the horizontal displacements of the column heads are critical. Materials are usually described via reduced compression strength; however, this work used crack propagation studies, which are more appropriate for concrete structures.

Direct studies of sulphate ingress involving concrete sampling and chemical groundwater analysis or the execution of non-destructive testing in the case of sulphate burns are specific and difficult to perform [1]. The motivation to present the reliability assessment of concrete columns grew from the evaluation of real foundation cases. Static load tests are performed for inspection of new settled columns and piles and treated as passing or acceptance tests. The rules of conducting the load test and its interpretation are described in [2, 3]. Lifetime displacements are difficult to measure in geotechnical implementation for complex interaction in structures with substrates, and numerical modelling is primarily used here especially for periodically aggressive environments.

The problematic foundations were located in areas containing substrates composed of non-bearing sediments and seasonal fluctuations in groundwater levels. The propagation of cracks and material degeneration caused chemical aggression.

A one-dimensional random field was associated with the analysed column via the non-linear stiffness of subsoil. The task was modelled numerically using the finite element method (FEM) with an elastic material for CMC subjected to elasto-plastic material stretched on one dimensional random field along with forces based on the *p-y* method. The numerical variant of the task was previously presented in [4, 5] for independent time conditions without deterioration.

The mechanical tasks were calculated independent of the sulphate progression into material. Chemical influence of deterioration was connected via the mechanical behaviour of the crack depth over a two-step approach. This was used as follows: the first was a stationary process with respect to the limited horizontal load of the column profile. Second, the phenomenon of pure concrete deterioration was supposed to be a time-dependent probability process. The column coupling with homogeneous soil mechanics and behaviour was used as a probabilistic method based on random field theory on soil mechanics as described by many previous authors [6–8].

The inverse Discrete Fourier Transformation DFT^−1^ based on white noise was used to generate the Gaussian Random Field (GRF) method and is wide spread in other technical branches [9] especially in signal analysis. This special procedure was used to protect the generated random fields from any case of unrealistic effects of long distances between correlated points (in a semi-variogram chart). Here, correlation functions were introduced with space variability in soil [10, 11]. The concrete sulphate aggression was described as orthogonal to a column external surface by two elements uncorrelated with each other random variables. The process was described for concrete elements where the crack depth from the sulphate ingress was synonymous with a loss in the volume of the material based on numerical solutions in a column cross-section.

## 2. Sulphate aggression

### 2.1. Description of the environment aggressiveness

The European Standard [12] presents the impact of the environment on concrete as a class exposure. The classification does not provide computational tools capable for solving ordinary engineering problems [13]. According to the National Code, concrete can be subjected to more than one environmental impact and described in several classes simultaneously. The separated exposure classes are as follows:

X0—class exposure in the absence of concrete threats from aggression or environmental corrosion,
XC—exposure class due to risk of carbonation,
XD—exposure class of the concrete due to risk of corrosion caused by chlorides not coming from seawater,
XS—exposure class of the concrete due to risk of corrosion caused by chlorides from sea water,
XF—exposure class of concrete considering the impact of freezing and thawing alternatively,
XA—exposure class of the concrete due to all other chemical aggression (including sulphate ingress).

Individual classes of chemical aggressiveness XA correspond to the concentration of ions, which allows them to be separated into additional subclasses in Table 1.

**Table 1:** Exposure classes XA and limiting values for exposure classes for chemical attack from natural soil and groundwater.

	Chemical characteristic	XA1	XA2	XA3
Ground water	SO_4_^2-^ mg/l	≥ 200 and ≤ 600	≥600 and ≤3000	≥ 200 and ≤6000
Soil	SO_4_^2-^ mg/kg^3^ total	≥ 2000 and ≤ 3000*	>3000* and ≤12000	≥ 12000 and ≤ 24000

* the 3000 mg/kg limit shall be reduced to 2000 mg/kg where there is a risk of accumulation of sulphate ions in the concrete due to drying and wetting cycles or capillary suction

### 2.2. Changes in the properties of concrete under the influence of sulphate ingress

Tow post-failure cases with significant engineering construction are presented. The key conclusion is that a drastic change in the mechanical properties of the concrete is due to deterioration at a specific time. These cases create a point of reference for further studies. They present a framework for further investigation with a large area of uncertainty including both the mechanics of the task levels as well as the chemical composition of the water. They are further illustrated as a probabilistic issue; after defining the state, they are solved by reliability methods [14].

#### Case 1: Bogatynia (Poland 2003)

Part of a power station building was made with reinforced prefabricated concrete construction. It has been in service for 46 years. The foundation was made from concrete columns, located in a layer of silt-exposure class XD with an approximate thickness of 4.0 m below which there was the substrate of sandy clays. The results of the groundwater test suggested the presence of high levels of sulphate ions. The seven concrete samples had 70% strength in relation to the baseline of C16/20. There was a significant reduction in the concrete strength caused by the chemical corrosion activated by groundwater filtering into silt. Samples were taken from a trench layer of the column 5–35 mm deep (the column was completely fissured).

#### Case 2: Gdansk (Poland 2012)

**Fig 1:**
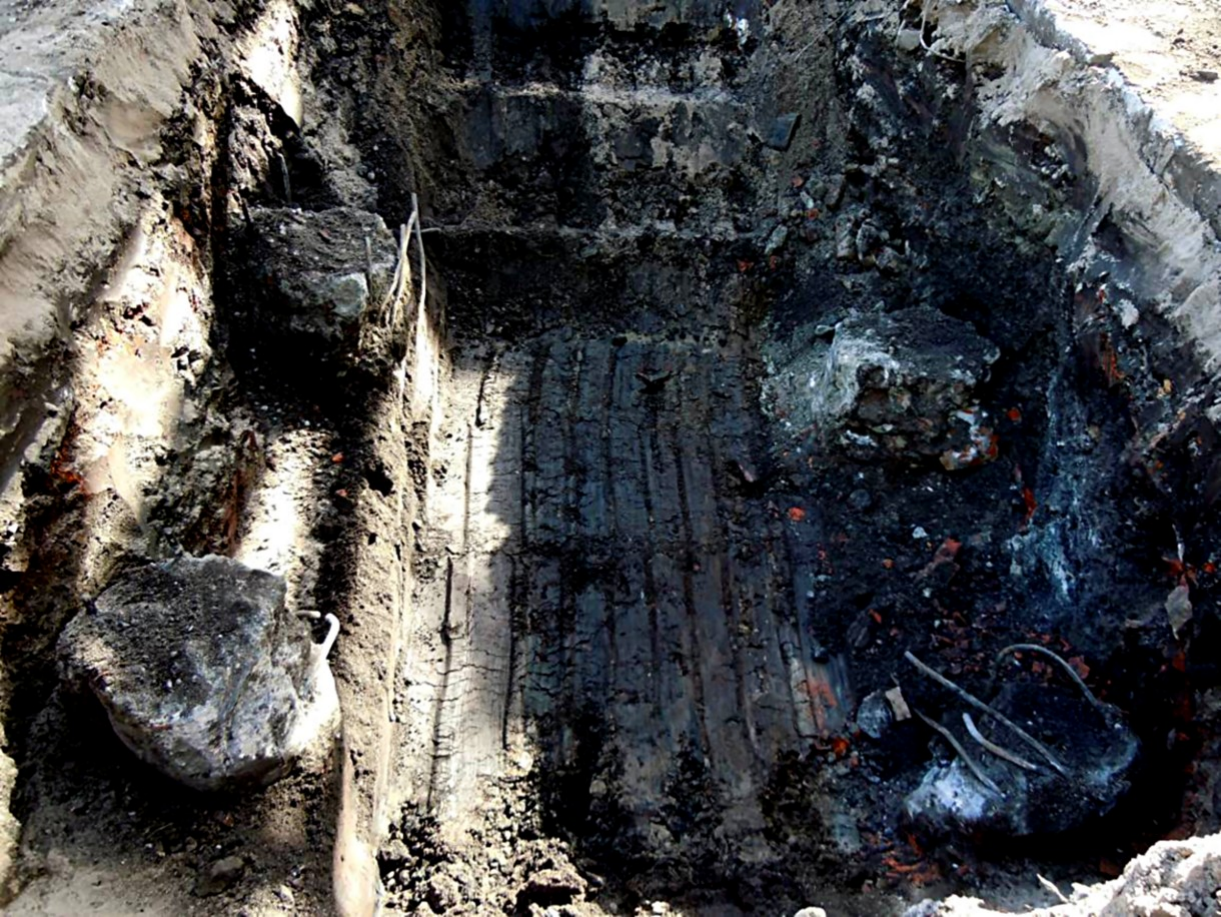
View of corroded column heads (Gdansk) including direct visual information about the process of corrosion.

Research was conducted on Franki cast-in-place piles after 33 years of performance on the manufacturing site. The 281 pile units remaining after a buy-and-hold investment remained unchanged. Considering the expansion of the factory plant, they analysed the possibility of rearranging a new foundation for a steam turbine block. The diameter of the column was 520 mm, and the length was 11 m in concrete class C16/20. Considering the expertise, 5 pits with a depth of 1.5–3.5 m below ground level unveiled a total of 21 columns (Fig 1). The fieldwork revealed that the diameter of the shaft columns and the depth of the material changes. The samples were measured via classical methods. Variations in the diameter of the column height and thickness were significant. The reinforcement was also corroded. The concrete was very porous. The samples were tested for the compressive strength of the concrete considering values lower than those designed in the range of 20–50%; the samples had a significantly reduced modulus (80% of the designed value). The water chemical analysis indicated a high concentration of sulphate ions.

### 2.3. Sulphate ingress in time-dependent formula as a reliability process

The cause of the destruction of concrete structures is sulphate digest. Chemical aggression leads to a loss of effective area of the concrete profile and decreased strength. This is a common cause of failure. Examples of aggressive environments [15] include the following general variants:

- relative humidity in the range of 60% and 98%,
- cycles of humidification and drying,
- cycles of freezing and defrosting; high carbon dioxide concentrations (e.g., seasonal de-icing pavements by salting),
- direct high concentration of chlorides or other salts (e.g., marine environments),
- high concentration of sulphates and small amounts of acids (e.g., sewer pipes or residual water treatment plants).

Structures such as CMC and piles are designed for a long service life, and hence the durability of the concrete plays a major role as shown in previous sulphate ingress examples.

**Fig 2:**
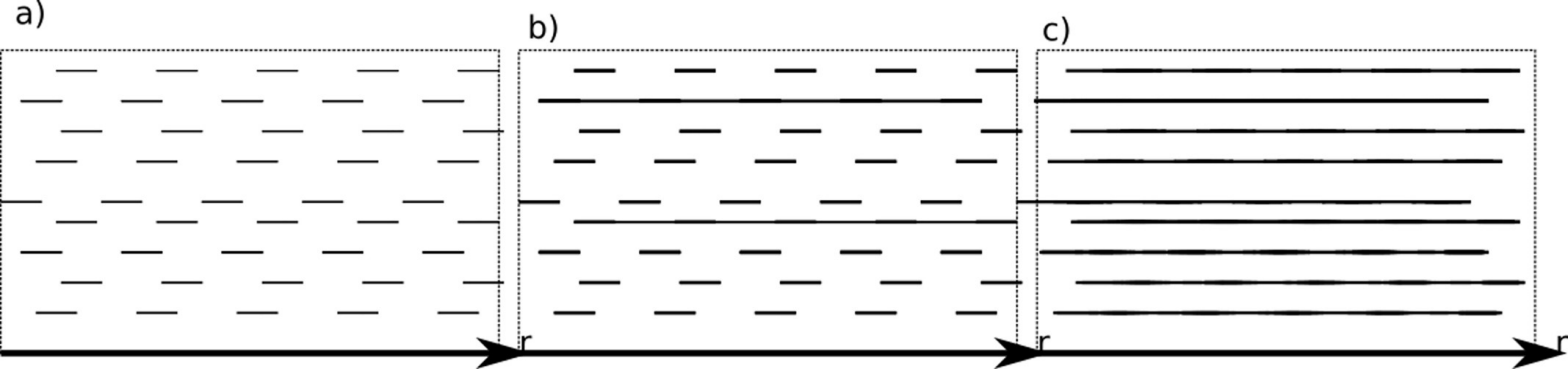
Transport ways for sulphate ions inside concrete trench (CMC) represents one dimensional regular grid in three percolations stages: a) concentration less than *K*_*th*_, b) concentration more than *K*_*th*_ and less than *K*_*dg*_, and c) concentration more than *K*_*dg*_.

The algorithm of deterioration used the percolation model [16, 17]. The micromechanical corrosion process was measured via progressive concrete crack density correlated with the concentration of sulphate ions. A high density of seams and their continuity is assumed—this allows us to apply the concept of percolation. To describe the diffusion coefficient, the permeability characteristics of the concrete were set to threshold concentrations (Fig 2):

- *K*_*th*_ is the conductivity percolation threshold; here, micro cracks are connected to form continuous channels that provide fluid flow (Fig 2B),
- concentrations below the conductivity percolation threshold *K*_*th*_ were studied without wasted water transport (Fig 2A)
- *K*_*dg*_ is the rigidity percolation threshold in which mutual influence of connected channels of microcraks causes a great loss of material stiffness (Fig 2C)

In this method, deterioration ingress was described by assumption *K*_*th*_ equal to *K*_*dg*_ calculated via the back-calculation method to numerical results. The porosity was taken from the literature [18] and [19] as follows:

$$\phi \prime =1-\frac{1+1.31\alpha \prime }{1+3.2w/c}$$ (1)

where α' is a degree of concrete hydration, and w/c ratio is the index of cement to water proportion from Table 2.

**Table 2:** Recommended limiting values for composition and properties of concrete.

	Exposure classes—Aggressive chemical environments		
	XA1	XA2	XA3
Max w/c ratio	0.55	0.50	0.45

The factor of diffusion after [20] with dependency to porosity is:

$$D(\phi \prime )=D_{0}(0.001+0.07{\phi \prime }^{2}+I(\phi \prime -0.18)1.8{\left(\phi \prime -0.18\right)}^{2})$$ (2)

where:

- *D* is diffusion coefficient dependent on porosity *φ*';
- ***I*** is similar to Heaviside's function; ***I*** is unity for *φ*' ≥ 0.18; in other cases ***I*** has a null value;
- *D*_*0*_ is diffusion coefficient for sulphate ions in water.

The model of the ingress of sulphate in concrete with respect to the function of time and depth using the Fick’s second diffusion law has got the following form in cylindrical coordinates:

$$\frac{\partial C}{\partial t}=\frac{\kappa_{s}^{*}}{r}\frac{\partial }{\partial r}\left(r\frac{\partial C}{\partial r}\right)$$ (3)

where:

- *C* is the concentration [mol/m^3^] in time *t* and radius *r* function;
- *κ*_s_^*^ is the diffusion coefficient in dimension of [m^2^ /s].

**Fig 3:**
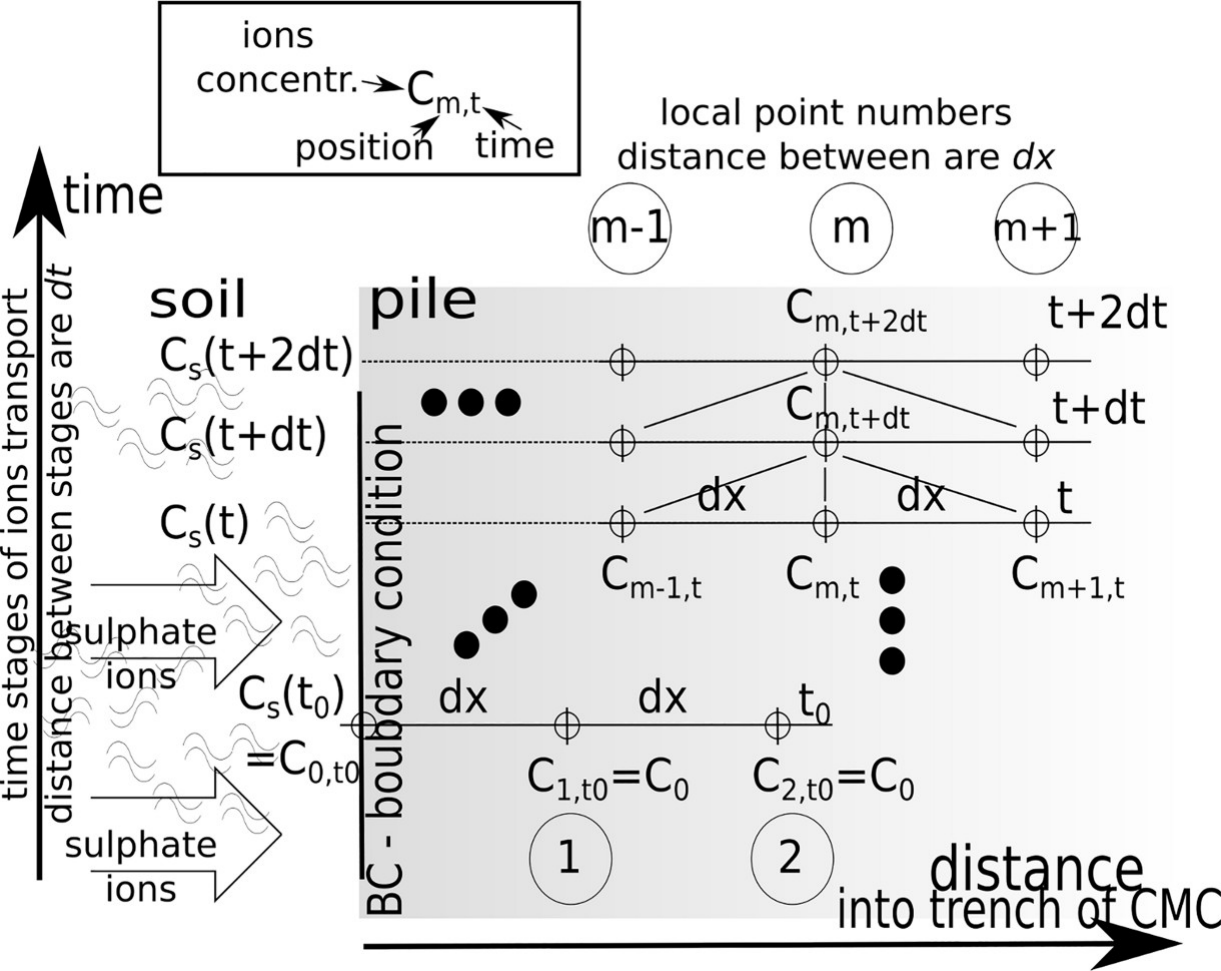
The procedure for numerical solution of transport issues for non-stationary boundary conditions and the functional dependence of the parameter and concentration *C*.

The solution of the differential Eq (3) in variable environmental conditions was obtained by the approximated Euler's procedure. The two first elements of Taylor's series were used to represent an unknown *C* function. The general idea of numerical solution of ions transport into a trench along with time is presented in Fig 3. This procedure used the discretisation of dimension *dx* and time progress *dt* for a depth of penetration in the one-dimensional process. The function *C* was represented as a rectangular matrix for columns {0,1,2, …*m*-1,*m*,*m*+1,..}*dx* and rows {0,1,2,3, …, }*dt* with the time function *C*_*S*_(*t*) as the boundary condition.

Infiltration of sulphate ions into concrete causes a decrease in pile mechanical properties. Similarly, crystalline compounds are destructed and porosity is increased. The rate of diffusion increases significantly after ions crossed the threshold concentration *K*_*th*_ [mol/m^3^]:

$$\kappa_{s}^{*}=\left\{ \begin{array}{c} {\alpha \;\kappa }_{s}\;K_{dg}=K_{th}\leq C\\ \kappa_{s}\;C<K_{th} \end{array}\right.$$ (4)

where:

- α is percolation factor recognized by back analysis [–];
- *κ*_*s*_ is a fixed nominal value of the diffusion coefficient (1, 2) [m/s^2^].

The failure area in the model is defined as the area where the sulphate ion concentration exceeds the value *K*_*th*_.

Eqs (1) and (2) create an axial symmetrical model that describes the degradation process of the concrete column.

To measure the degree of destruction *D*, we used a common concept:

$$D_{i}=1-\frac{\sigma_{i}}{\sigma_{0}}$$ (5)

where:

- *σ*_*i*_ is uniaxial compressive strength of concrete after time [MPa];
- *σ*_*0*_ is the initial uniaxial compressive strength of concrete [MPa].

Term *D’* is defined later [21] as:

$${D\prime }_{i}=1-\frac{r_{i}}{r_{0}}$$ (6)

Here strength on the related surface of unfractured concrete was replaced by the proportion of effective radius *r*_*i*_ to the nominal pile value *r*_*0*_. The effective value of radius is equal only in the virgin core radius *r*_*o*_ with ionic concentration less than the threshold *K*_*th*_.

**Fig 4:**
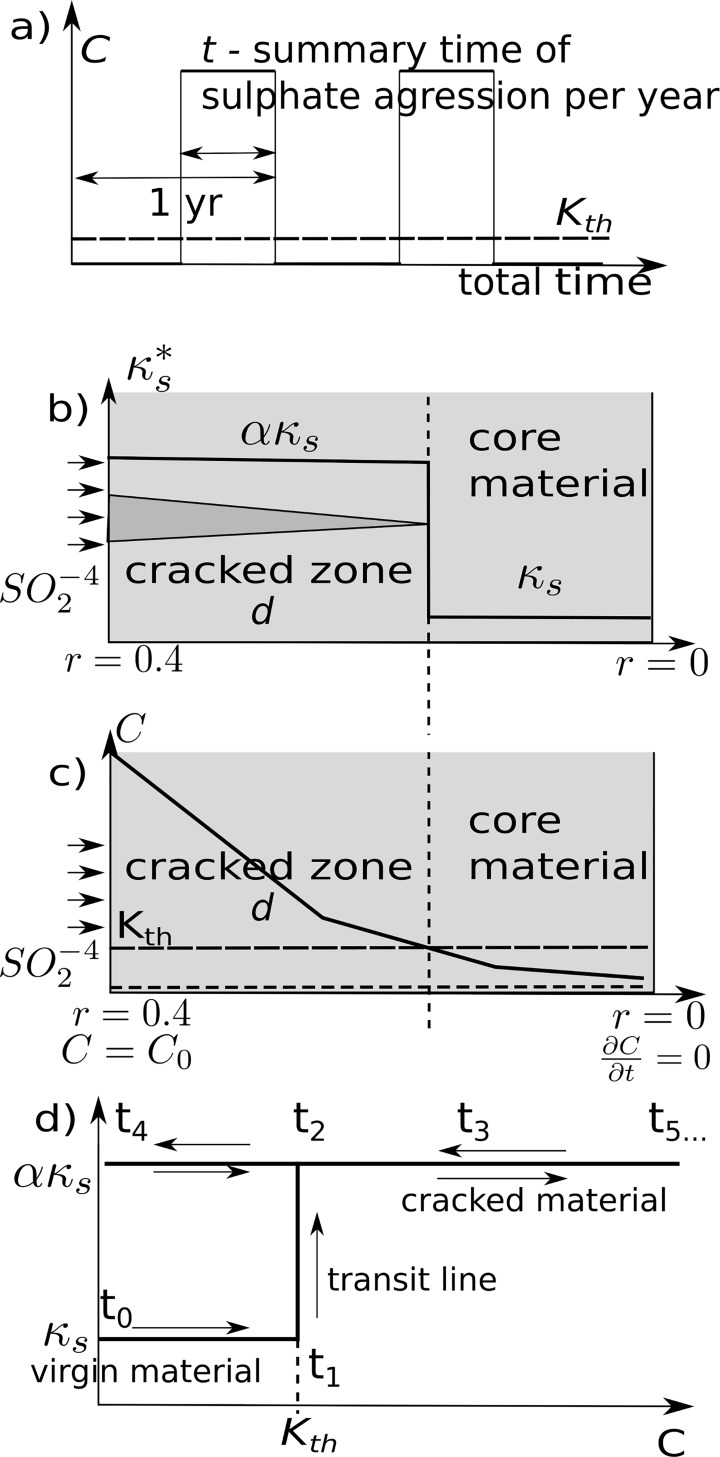
a) Boundary periodic load—ionic concentration was modelled as a periodic and discrete distribution; b) The area in which the concentration exceeds the threshold *K*_*th*_ is considered to be destroyed; c) Sketch of ionic concentration in the material with the boundary conditions; d) Path changes chart of the coefficient *k*_*s*_ with a specified hysteresis in time.

The boundary conditions and penetration process into pure concrete is presented in Fig 4.

The task was solved in a fitting procedure where *α* and *κ*_*s*_ coefficients were calibrated in FlexPDE to the results of the experiment conducted after laboratory experiments after work [22] ions concentrations of 10% and 20%.

These conditions correspond to SO_2_^-4^ concentrations of 1.052 g/cm^3^ (10%) and 1.106 (20%) g/cm^3^, which respectively were represented by 1.0951 mol/dm^3^ and 2.3027 mol/dm^3^. They represent the boundary conditions for *r*_*0*_.

The process of concrete cubic brick destruction for the 10% and 20% ion concentrations was solved (Table 3) with calculated coefficients *α* = 2.017, *κ*_*s*_ = 3.09 10^−6^ [m/s^2^].

**Table 3:** The results of experimental research based on [22] and relative error for the proposed model.

Time [month] [60*60*24*30 s]	*D’*_*i*_ for 10% ions concentration [–]	Relative error of model to experimental results [%]	*D’*_*i*_ for 20% ions concentration [–]	Relative error to experimental results [%]
0	0.000	0.0	0.000	0.0
1	0.032	0.8	0.049	1.1
2	0.061	1.9	0.087	1.3
3	0.080	2.0	0.123	-0.3
4	0.120	0.0	0.162	-2.2
6	0.158	-1.8	0.201	-2.1
8	0.175	-1.5	0.232	-1.2
10	0.201	-2.1	0.258	-1.8
12	0.224	-2.4	0.277	-0.2
15	0.250	-3.0	0.298	-1.0

The relative errors between experimental and model-based results are below 3.0%, which suggests that the model describes the process of sulphate ingress.

## 3. Mechanical model

The stiffness of the soil was assumed to be dependent on depth *z* under terrain as follows:

$$G_{p}(z)=G_{srf}+{\left(A_{w}z\right)}^{k}$$ (7)

Here, *Gsrf* is the stiffness at the ground surface; (*z* = 0), *z* is the depth along a column; *Aw* and *k* are coefficients for the fitting function to soil profile data.

The limit state in cohesionless soil at depth *z* is described by Mohr- Coulomb's law by passive earth pressure:

$$p_{\lim}(z)=\frac{1+\sin\phi }{1-\sin\phi }\gamma z$$ (8)

**Fig 5:**
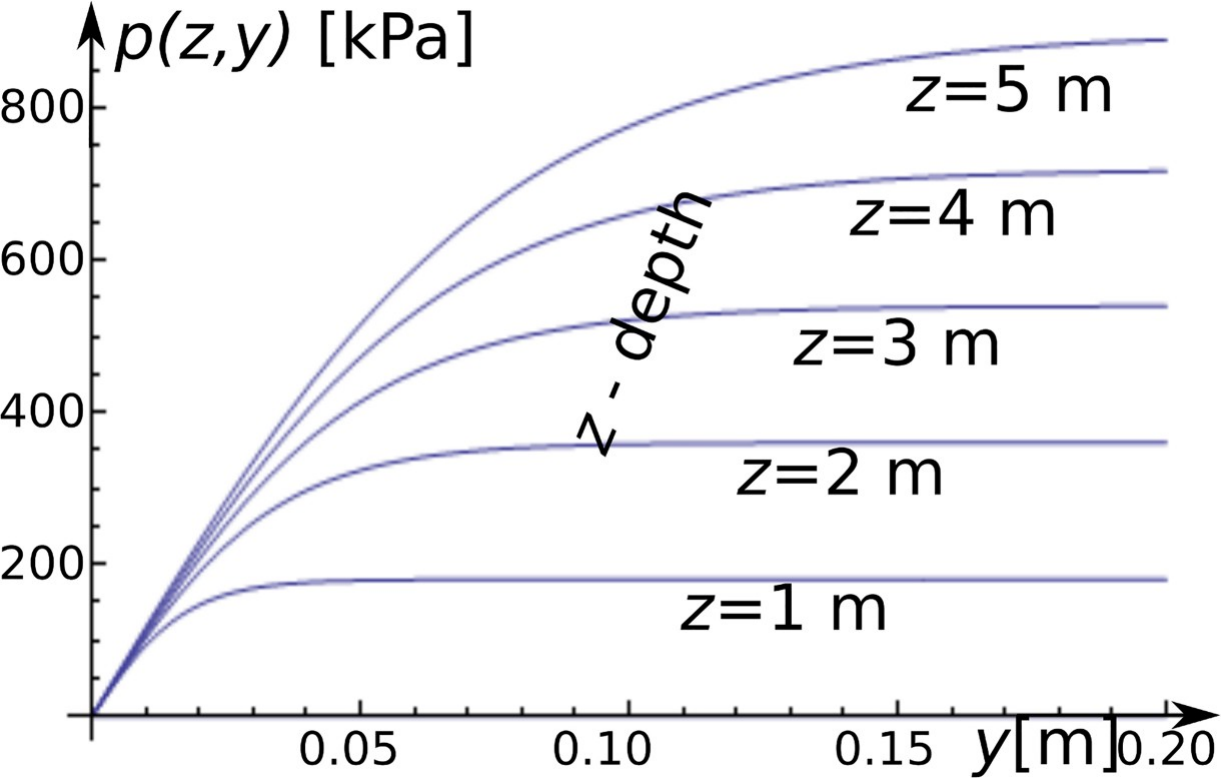
The *p-y* curves. The horizontal axis is horizontal displacement of segment *y* [m], and the vertical axis is the soil response [kPa] at a soil depth *z* = {1, 2, · · ·, 5} [m] for the selected curves.

Here, *γ* is soil volume weight, and *φ* is internal friction angle of soil. In segments of column with length *dl*, the soil stiffness has the following form:

$$k(z)=HG_{p}(z)dl$$ (9)

and related *p-y* curves described by a hyperbolic tangent have the form:

$$p(z,y)=A_{p}p_{\lim}(z)\tanh\frac{k(z)y}{A_{p}p_{\lim}(z)H}$$ (10)

where added *H* is the column diameter with nominal value equal 400 mm, *y* is a deflection of segments in the horizontal direction, and *A*_*p*_ is fitting parameter. Fig 5 presents selected curves *p-y* in depth function.

The finite element stiffness along of the column was set as the ideal elastic material for each of beam segments. Considering a depth of elements in the latter part of the calculation, the beam segments were related to a zone covered by sulphate ingress. A solution was obtained iteratively due to the non-linear functions of *p-y*.

**Fig 6:**
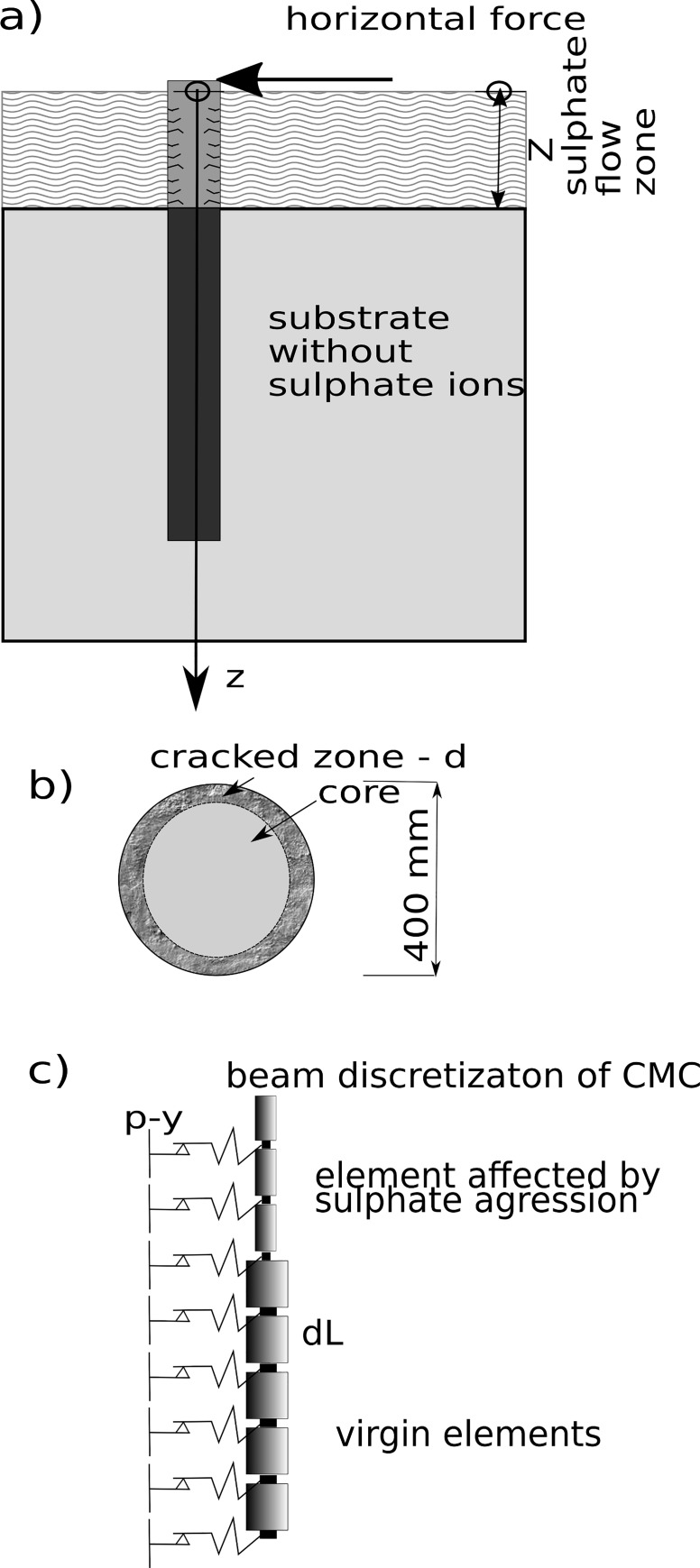
a) Diagram of the task with the model of relation between column and soil; b) the cross-section of the column in the area of sulphate aggression and: c) CMC beam discretisation scheme.

Calculations suggested that a horizontal constant force of 50 kN was used on the column head (Fig 6A). The maximum size of the zone of sulphate aggression is defined as the distance from the ground surface (z = 0) to a lower point of the groundwater aggressive stream *Z*. The fissured layer has a maximal thickness of *Z* = 5.0 m. The elastic modulus for pure concrete (virgin state) in the initial time was equal to 30 GPa. We assumed a complete loss of load capacity in the fissured layer of concrete as a result of combined factors: 1) the tensile stress above a threshold initiating cracks and 2) a rapid increase in porosity. The nominal diameter of column CMC was *r*_*o*_ = 400 mm and its length is *L* = 10 m. The mechanical parameters of the analysed substrate are described by the following: *Aw* = 100 [–], *k* = 1.2 [–], *φ* = 30*o*, *Gsrf* = 10 MPa. The number of finite elements (*dL* on Fig 6C) along the beam trench was equal to 20 pieces, and the bulk density of the soil was *γ* = 20 kN/m3. The mechanical tasks were studied for the impact of progressive chemical deterioration in increments of 5 mm. We systematically reduced the diameter of the column core (the maximum range of down-hole corrosion amounted to 80 mm). The head displacement was checked for all combinations of depth *d* fissured zones.

## 4. Reliability task for columns under lateral load

### 4.1. Sulphate deterioration as a non-deterministic process

The statistical and probability description of deterioration was performed on 107 drawing number for progressive sulphate ingress with two selected random variables (Table 4). Both random variables *X* were defined as a symmetrical beta PDF by minimal min[X] and maximum max[X] values where mean *μ*(*X*) = 0.5(min[X] + max[X]) with the following shape parameters [23, 24]:

$$\alpha =\beta =\frac{1}{8}\left(\frac{1}{var{\left[X\right]}^{2}}-4\right)$$ (11)

**Table 4:** Random variables that describe the concrete sulphate aggression process and their descriptions of probability distributions.

*random* variable *[*X*]*	Probability distribution	Var [X]	Min [X]	Max [X]
*C*_*0*_ (ion concentration at column trench)	beta symmetrical	0.15	10[%]	20[%]
*λ* (time of sulphate ions attack per year)	beta symmetrical	0.15	0[days/year]	12[days/year]

Periodically occurring sulphate aggression in the vicinity strata of the column trench assuming a threshold *K*_*th*_ (Eq (4) and Fig 4) for the diffusion coefficient. This system was modelled as an aggressive environment with a relation = *λ t*_*total_time*_, where:

- *t*—aggregate time of occurrence of the aggressive environment;
- *t*_*total_time*_—lifetime of construction;
- *λ*—coefficient of accumulated time of corrosive environment to the total lifetime with a range: [0, 1].

Periodic ingress of sulphate into concrete reduced the thickness of the column over time. This dependence as a function of the log-normal probability distribution was fitted to the result of the deterioration depth independent of mechanical deformation of the column.

The chemical sulphate ingress into the column is approximated by a probability density function based on histogram results with relevant parameters μ(*t*_*0*_) and *σ(t*_*0*_*)* for *t*_*0*_ in range of {15, 20, …,50} years.

The results were obtained as a set of the eight histograms prepared for each 5-year intervals; they describe the sulphate ingress over time. A log-normal probability distribution was chosen for the histograms, and the parameters were separately adjusted to each of the histograms. They are shown in Table 5 as list of CDF parameters *Φ*_*d*_(*μ*(**t**),*σ*(**t**)) where *t* represents discrete time.

**Table 5:** Chemically destroyed depth *d*_0_ as a reliability process for parameters of log-normal distribution (*μ*, *σ*) with time influence.

Time [years]	*μ* [mm]	*σ* [mm]
15	-3.839	0.230
20	-3.640	0.236
25	-3.493	0.237
30	-3.379	0.243
35	-3.285	0.246
40	-3.202	0.248
45	-3.133	0.254
50	-3.066	0.254

This approach is named the Modified Response Surface (MRS) method and was evaluated according to the classical Response Surface (RS) method widely descripted in [25]. In the RS method, discrete values of *RS*(***x***) are approximated by a continuous function as follows:

$$RS(x)=RS^{\prime }(x)+E(x)$$

where *RS'*(***x***) is an approximate function including high dimensional model representation [26], polynomials, neural networks or wavelons. Term E(x) is an error of approximation; ***x*** is a random vector of mechanical properties (or loads $x\in \Omega \subset R^{N}$) where *N* is dimension of subspace Ω domain.

The RS method was developed for cover by a continuous approximated function. The hidden relations were added to the process where only limited number of data points are unveiled. However, the RS classical method has some weaknesses—especially close to the discontinuity of the original function. The error values from fitting are transferred and multiplied to the probability calculation. When the task has more dimensions in hyperspace of random variables, then the discreteness points are hardly detectable. The larger sample of data was reached in the task both for the mechanical system and for the chemical process. The MRS method is a concept based on a discrete number of CDFs related with variables. The approximation occurs at the CDF distribution level. The result is continuous in the time domain function of Φ'_*d*_(*μ*'(*t*),*σ*'(*t*)):

$$\mu \prime (t)=\mu (t)+\varepsilon_{\mu }(t)\;\mathrm{and}\;\sigma \prime (t)=\sigma (t)+\varepsilon_{\sigma }(t)$$

where *μ*(*t*), *σ*(*t*) are approximate functions. The ε_σ_(t) and ε_μ_(t) are errors of approximation. The fitted CDF Φ_*d*_*(μ*(*t*),*σ*(*t*)) was prepared by the least square method LSM based on polynomials.

Values of parameters of log-normal CDF: Φ_*d*_(*μ*(*t*),*σ*(*t*)) were estimated in time *t* as a function of random variables for crack depth *d*(*t*) described by parameters:

$$\mu (t)=-4.445+0.04762\;t-4.062\cdot 10^{-4}t^{2}$$ (12)

$$\sigma (t)=0.2194+7.671\cdot 10^{-4}t-2.05010^{-7}t^{2}$$ (13)

**Fig 7:**
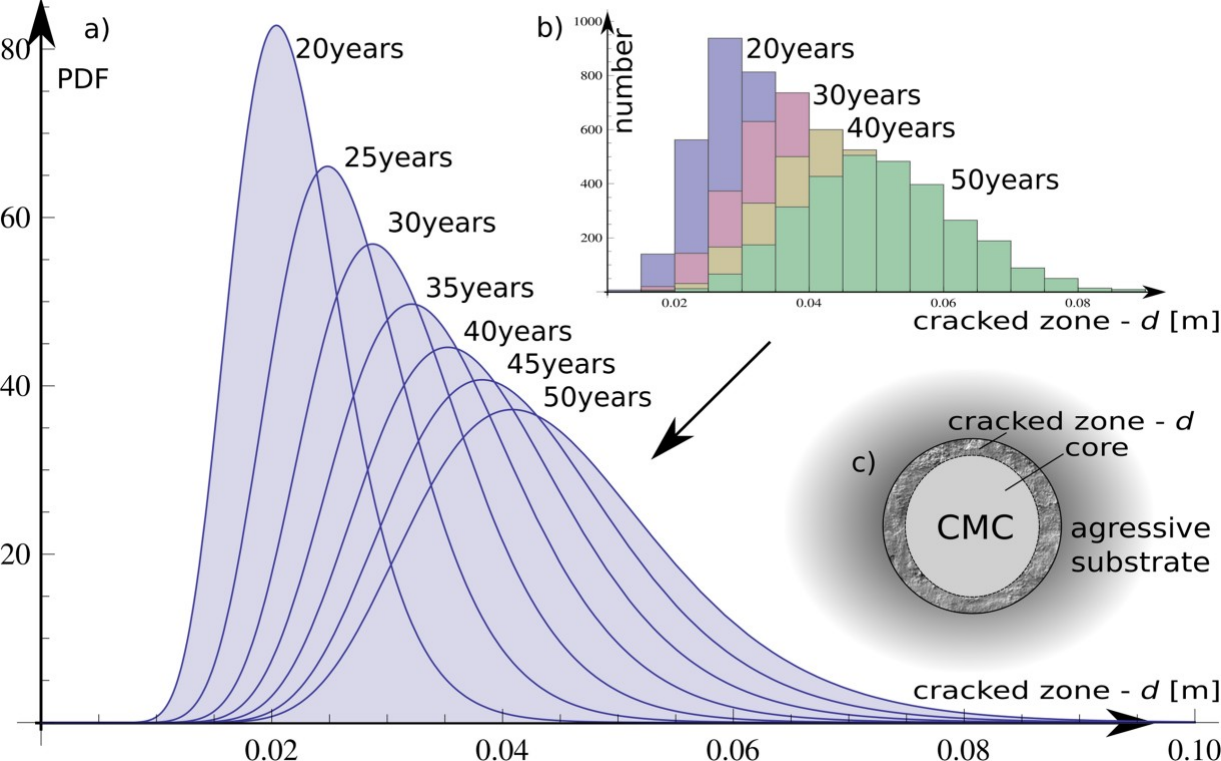
Deterioration depth over lifetime as a result of probability separated calculations: a) fit log-normal PDFs; b) histogram from raw results in every 10-year period.

The fitting results are illustrated in Fig 7. Throughout the process, the resolution of a 5-mm crack reaches the depth distribution fit via a log-normal PDF (i.e., for 30 and 50 years, presented by cross-sections). The results are shown also in Table 5.

### 4.2 Substrate parameters

The substrate material was described by using a random field $\bar{\phi }(z)$ of friction angle with a vertical fluctuation scale *θ* = 2.5 m along the column edge. The random field $\bar{\phi }(z)$-modified stiffness of the substrate (6) is as follows:

$$G_{p}(z)=G_{srf}(1+0.1\bar{\phi }(z))+{\left(A_{w}z\right)}^{k}$$ (14)

The change of the stiffness was also correlated with the passive ground pressure:

$$p_{\lim}(z)=(\left(1+\sin\left(\phi +\frac{\pi \bar{\phi }(z)}{180}\right)\right){}^{2}\gamma z$$ (15)

**Fig 8:**
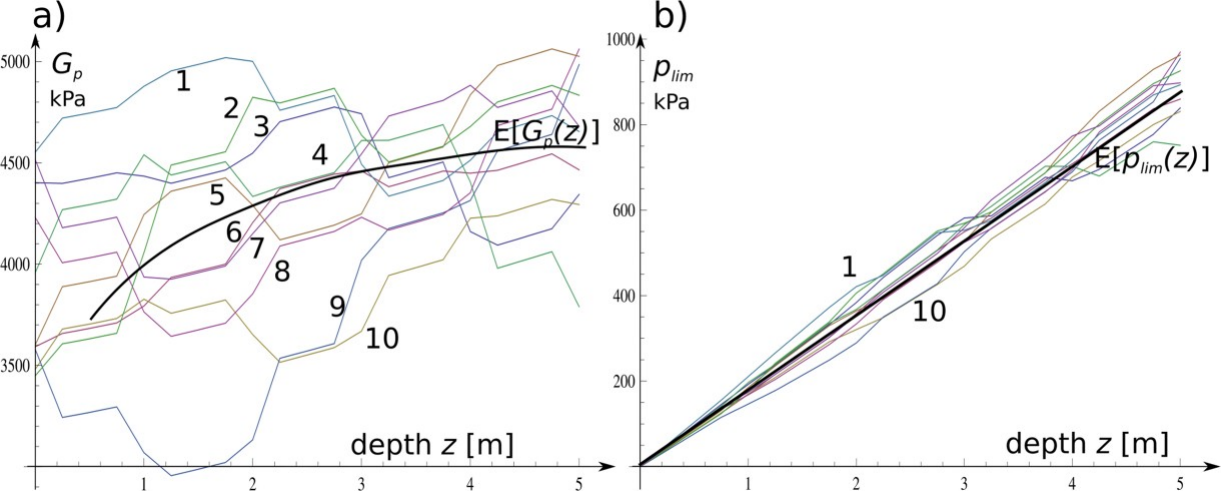
a) Example graphical presentation of *Gp—*10 drawings at a depth of z; b) *pli*m is presented by 10 drawings also with the depth function. Both graphs are prepared for the identical scale fluctuation equal to *θ* = 2.5 m.

The impact of $\bar{\phi }(z)$ on both variables is characterized in Fig 8 with charts prepared for a set of random realizations.

### 4.3 Relation of mechanical and flow sulphate ions models

**Fig 9:**
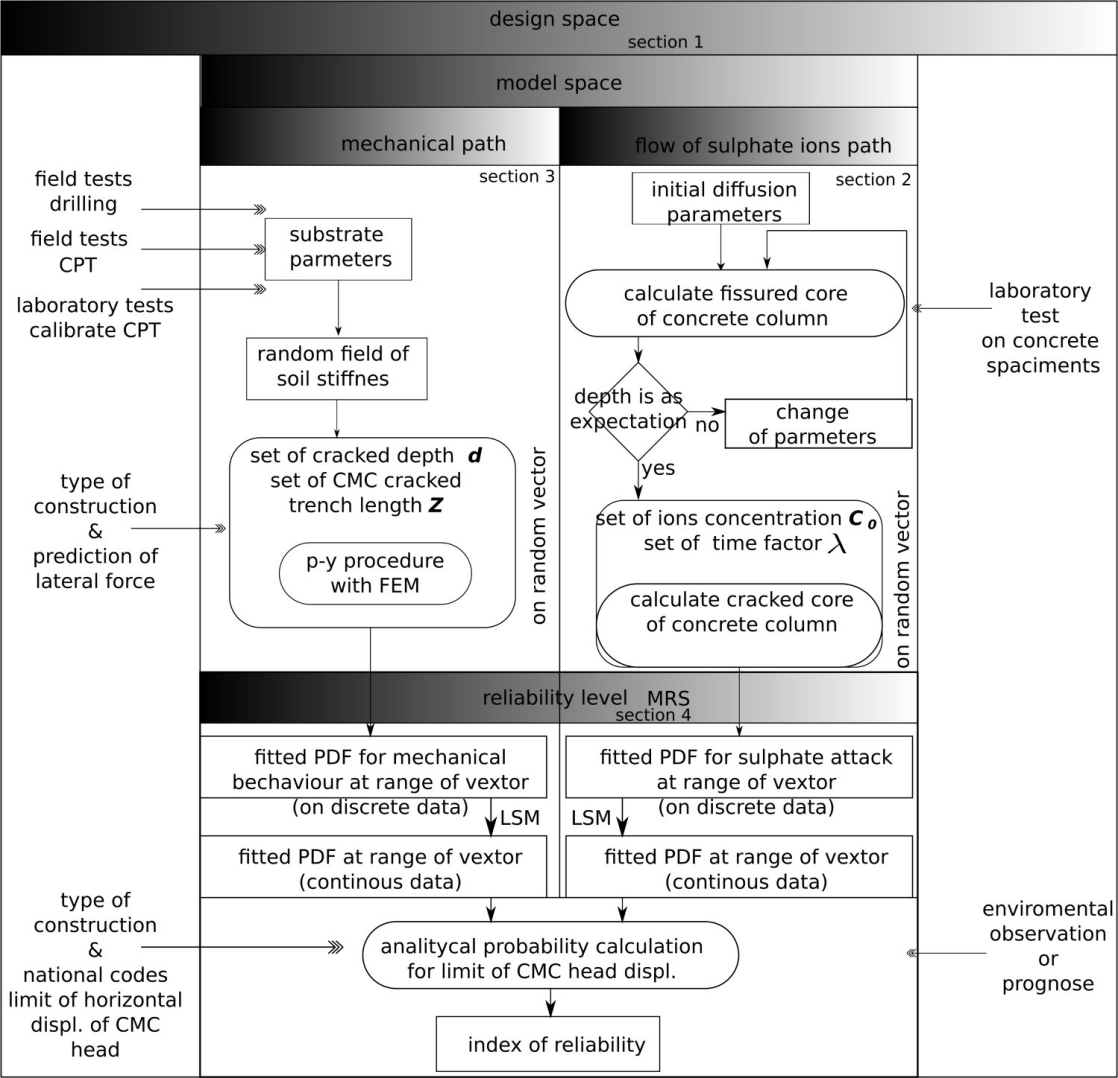
Flow diagram of procedures for describing and modelling object under environmental load.

Fig 9 details the main idea for assessing the reliability index *β* for the existing or desired structure in aggressive environments. This diagram links to sections and is a manual guide for the entire method. The area from both border sides of the core diagram represents data from extended acquisition. A column influenced by lateral force and ion diffusion is presented in specified sections of the diagram; the methods were repeated independently in Section 2 and Section 3.

The deterioration algorithm estimates the fissured zone as a function of ion concentration and time. The set of discrete solutions from Section 3 defines the fissured core over lifetime as a result of separated calculations. The solutions from Section 2 appeared as head displacements. All of these were prepared for all possible combinations of fissured trench of column length *Z* with depths *d*; this allowed conversion of discrete data by fitting to PDF.

The next calculations are the probabilistic level of the task. Section 4 shows that the value of the fissured zone is used in the mechanical model of the column to determine the head displacement and examine the probability of exceeding the acceptable value.

### 4.4 Results of the numerical calculations

The general algorithm to generate random fields is prepared via the literature [27] in Mathematica using the quick discrete inverse Fourier’s transform (DFT*−*1) for the direct construction of discrete Fourier’s transform (DFT) based on white noise. A random field with both a real and an imaginary part was constructed. Simulations and statistical tests RGF of the generator were prepared. The simulations were performed on a one-dimensional space with the power spectrum function [28, 29]:

$$\alpha (p)=\exp\left(-{\left(\frac{p}{\theta }\right)}^{2}\right)$$ (16)

Here, *p* is the distance between height points (lag), and the *θ* range scale fluctuations are assumed to be a constant in the task. The field was generated as a discrete object in the middle point of finite elements along with large margins equal to 5*θ*. This was cut to the field dimensions needed for *p-y* calculations. This procedure was used to reduce unrealistic effects in the semivariogram for long lags [30].

We show an example of the calculations that examined the results of exceeding the maximum displacement for the head of the CMC defined as serviceability failure state.

These calculations were performed according to the following scheme:

- generate the random field;
- calculate head displacement for all combinations of the polluted stream.

The fissured thickness was calculated by the sulphate deterioration progress in normal direction to trench CMC *d* = {0.000, 0.005, …, 0.080 } m and was combined with cases of active fissured zone depth (thickness of the aggressive stream around the CMC): *Z* = {0.00, 0.25, …, 5.00 m}. The assessment procedure of FEM iteration solutions was closed in most cases in less than 12 internal calculation steps for each set of mechanical parameters. The numerical experiment provided discrete histograms, which were fitted by log-normal probability distributions PDF with respect to their characteristics similar to the MRS method. The selected PDF functions are presented in Fig 8 with histograms in the background. The fitting of log-normal PDFs *Φ*_*disp*_(*μ*(*d*,*Z*),*σ*(*d*,*Z*)) for mechanical processes in cases of vertical displacement of the column head is as follows:

$$\mu (d,Z)=-5.4558-3.5162\;d+50.038\;d^{2}+0.2218\;Z+1.9030\;dZ-0.03813\;Z^{2}$$ (17)

$$\sigma (d,Z)=0.07638-0.36938\;d+2.6206\;d^{2}-9.006\cdot 10^{-4}Z+0.06177\;dZ+3.650\cdot 10^{-5}Z^{2}$$ (18)

where:

- *d* is thickness of fissured radius;
- *Z* is the depth of the fissured zone along the CMC trench.

**Fig 10:**
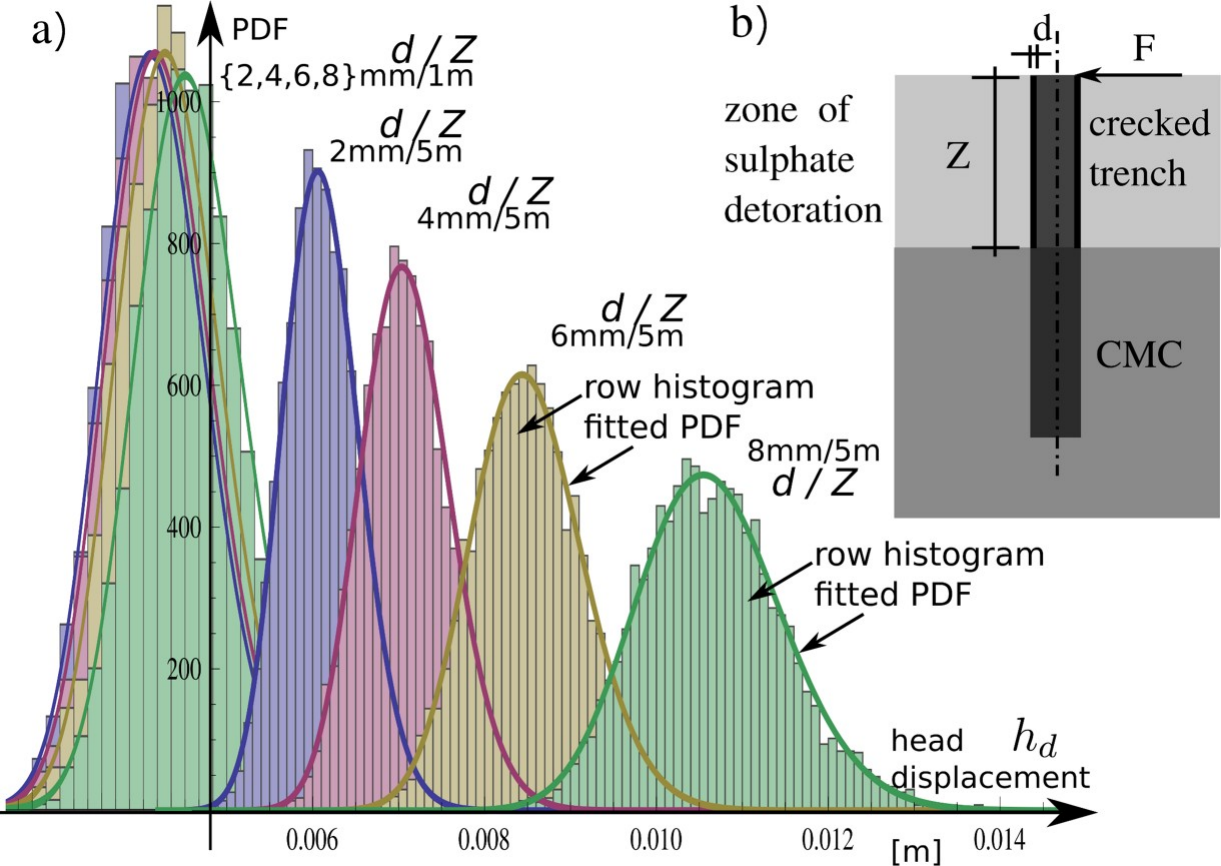
The selected log-normal distributions created for the results of column head horizontal displacement histograms for two combinations of analysed cases with 1- and 5-m thick fissured zones as well as for *d* = {2, 4, 6, 8} mm sulphate deterioration depths into the column skinned surface in each zone variant.

The PDFs for the mechanical part *Φ*_*disp*_ (μ(*d*,*Z*),*σ*(*d*,*Z*)) and for ingress of the sulphate front *Φ*_*d*_(*μ*(*t*),*σ*(*t*)) were used to directly study reliability calculations. The impact of the destructed zone dimensions for the reliability index is shown in Fig 10.

The two random elementary processes—sulphate ingress and head deflection of structure—are related together via probability descriptions as follows:

$$\Phi (t)=\Phi_{disp}(\mu (d,Z),\sigma (d,Z))\Phi_{d}(\mu (t),\sigma (t))$$ (19)

**Fig 11:**
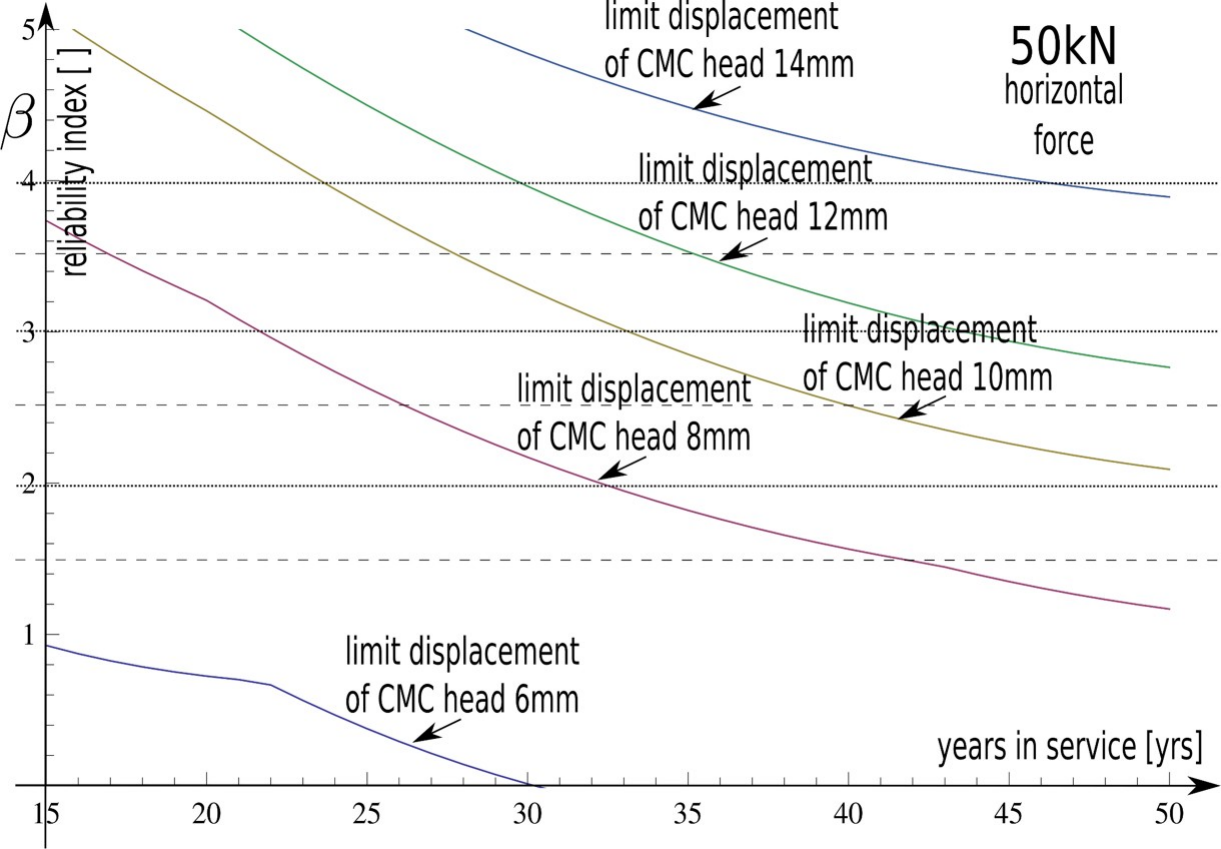
The function of change of reliability index *β* over time for different values of column head displacement limits *hdult* = {6, 8, 10, 12, 14} mm.

This results in the values of *β* presented in Fig 11 for different values of column head displacement limits *h*_*d*_^*ult*^ = {6, 8, 10, 12, 14} mm.

## 5. Discussion

The main aspect that limits the lifetime of analysed geotechnical constructions was lateral displacement of column heads. The reliability results are presented for discrete collections of allowable limits of the serviceability state for the following: *h*_*d*_^*ult*^ = {6,8,10,12} mm. The values are determined by standard limitations used for bridges. The support system is damaged when it reaches or exceeds the serviceability state. In practice, the allowable value of the horizontal head displacement is less than 10 mm. Here, the safe lifetime was over 50 years, and the corresponding reliability index is two: this is lower than the standards. The ISO Standard [31] for serviceability limit states recommends adopting reliability indices dependent on the consequences of possible failure and the cost of a repair. The value of the reliability index can be much higher when highly ‘responsible’ structures using bearing capacity limit states are designed. To ensure greater value of reliability, it is necessary to increase the CMC diameter or use a higher grade of concrete. Alternative methods to prevent CMC against sulphate attack include covering with low permeability soil, sodium silicate, or resins. The values of the reliability indexes as a function of year are presented in Fig 11 via a convenient operating symbol of the reliability index *β*. Some general phenomena are coherent with intuitive approaches where a decrease of limit restriction results in higher values of the reliability index.

An increase in the depth of sulphate ingress into concrete leads to lower *β* values. The derivative effect is an occurrence of the falloff associated with the depth of sulphate attack *d* in a column. Eq 17 and Eq 18 parameters are associated with *d*^*2*^. They are much higher than others, and they determine the probability of failure. This phenomenon has origins in column mechanics coupling with the substrate. Fig 10 shows that PDFs have two calculated far situations for a range of attacked trench of CMC (*Z* = 1 m and *Z* = 5 m). The impact of sulphate digestion on vertical length impacts the mechanics of the beam divided into two parts: both have different inertia moduli.

The relationship between *Z* length and the reliability index is inverse proportionality: This observation agrees with field experiments. Passing time decreased the reliability of construction likewise to previous phenomena. The impact of time is shown in Fig 7, Eq 12, and Eq 13. The general dependency is clear—longer exposure to periodic wetting in aggressive environments lowers the reliability and increases crack depths along the column. We observe unexpected effects including quicker degradation of the column in the first 30 years as well as a decrease in crack progression in the next 20 years (Fig 7 and Fig 11). This is because there are longer ways of ion transport way into the healthy core of the column. The influence of time on the structural safety is detailed here. The results confirmed the research assumptions.

## 6. Summary

This work created a response to the lack of general guidelines beyond design and classification rules for controlled modulus columns. The complexity of the problem for lateral loads is described as follows:

- an environment with progressive sulphate ingress;
- material loss in structural composition along with destruction processing;
- random mechanical parameters of the substrate.

This work evaluated the reliability of specific types of embankment support in harsh environments. It can be extended to other concrete elements including piles or supporting walls during material degradation. Fig 9 shows support and guide for engineers via a flow chart. We also made some simplifying assumptions to emphasize pre-selected effects:

- the random field that modelled the soil properties was one dimensional;
- the model of soil-column interactions needs future study for full representation as a FEM 3D model;
- only pure concrete material restricts sulphate deterioration.

The processes shown here for the lifetime of the construction considers time as an important operating factor for engineers. It reduces the level of confidence for existing objects. The use of ground improvement and the CMC system offer cost and time savings and a more sustainable solution for constructing foundations on building sites with poor quality soils versus more traditional solutions. This approach measured sensitive complex systems reliability for time-dependent processes. The tool can answer the basic questions that designers have about the relationship between the limitations of head displacement conditions, time, and safety. The main calculation modules in the article include the random field generator GRF, the chemical aggression process, and the *p-y* FEM.

## Supporting information

S1 File FlexPDE procedures for sulphate attack calibration. (PDE)

S2 File Pile in FEM embedded in the soil. (NB)

S3 File Scheme of *p-y* method. (PS)

S4 File Results of *p-y* procedures. (CSV)

S5 File Results of FEM procedures. (CSV)

S6 File AME certificate. (PDF)

## References

[1] Mori Y.; Ellingwood B. R. Reliability based service life assessment of aging concrete structures. J. Struc. Eng. 1993, 119, 1600–1621.

[2] Rybak J. Some Remarks on Foundation Pile Testing. IOP Conf. Series: Materials Science and Engineering 2017, 245, art. 022092, 10.1088/1757-899X/245/2/022092

[3] Muszyński Z.; Rybak J. Horizontal displacement control in course of lateral loading of a pile in a slope. IOP Conf. Series: Materials Science and Engineering, 2017, 245, art. 032002, 10.1088/1757-899X/245/3/032002

[4] Bauer J.; Kozubal J.; Puła W.; Wyjadłowski M. Application of HDMR method to reliability assessment of a single pile subjected to lateral load. Studia Geotechnica et Mechanica 2012, 34, 37–51.

[5] Kozubal J.; Puła W.; Wyjadłowski M.; Bauer J. Influence of varying soil properties on evaluation of pile reliability under lateral loads. Journal of Civil Engineering and Management 2013, 19, 272–284.

[6] Ching J.; Lin C. Probability distribution for mobilized shear strengths of saturated undrained clays modeled by 2-D stationary Gaussian random field—A 1-D stochastic process view. Journal of Mechanics 2014, 30, 229–239, 10.1017/jmech.2014.9

[7] Ching J.; Phoon K.; Kao P. Mean and variance of mobilized shear strength for spatially variable soils under uniform stress states. Journal of Engineering Mechanics 2014, 140, 487–501, 10.1061/(ASCE)EM.1943-7889.0000667

[8] Emir Ahmet Oguz; Nejan Huvaj; Griffiths D.V. Vertical spatial correlation length based on standard penetration tests. Marine Georesources and Geotechnology 2018, 10.1080/1064119X.2018.144318

[9] Knill O. Probability Theory and Stochastic Processes with Applications; Overseas Press, New Delhi, India, 2009, ISBN 81–89938–40–1.

[10] Jaksa, M.; Kaggwa, W.; Brooker, P. Experimental evaluation of the scale of fluctuation of a stiff clay. In Proceedings of the 9 th Australia New Zealand Conference on Geomechanics, Auckland, Australia, 2004.

[11] Jamshidi C.; Oloomi D. New method for estimation of the scale of fluctuation of geotechnical properties in natural deposits. Computer Methods in Civil Engineering 2010, 1, 55–64.

[12] European Standard 206–1 Concrete—Part 1: Specification, performance, production and conformity. European Committee for Standardization, 2005.

[13] Low B. K.; Phoon K. K. Reliability based design and its complementary role to Eurocode 7 design approach. Computers and Geotechnics 2015, 65, 30–44, 10.1016/j.compgeo.2014.11.011

[14] Kozubal J.; Szot A.; Steshenko D. Improved road embankment loess substrate under earthquake hazards In Underground infrastructure of urban areas 3; Madryas C.; CRC Press, Taylor & Francis Group 2015; pp. 53–62, ISBN 9781138026520—CAT# K24213.

[15] Emilio B.-A.; Mauricio S.-S.; Alaa C.; Moema R. Coupled reliability model of biodeterioration, chloride ingress and cracking for reinforced concrete structures. Structural Safety 2008, 30, 110–129.

[16] Basista M.; Weglewski W. Micromechanical modeling of sulphate corrosion in concrete: influence of ettringite forming reaction. Theoretical and Applied Mechanics 2008, 35, 29–52.

[17] Węglewski W.; Basista M. Chemically Assisted Damage of Concrete: A Model of Expansion Under External Sulphate Attack. International Journal of Damage Mechanics 2009, 18, 155–175.

[18] Pommersheim J.; Clifton J.R. Sulfate attack of cementitious materials: volumetric relations and expansion, NISTIR 5390, National Institute of Standards and Technology, Gaithersburg, MD, 1–19.

[19] Skalny J.; Marchand J;. Ivan Odler. Sulfate attack on Concrete. CRC Press: Boca Raton Florida, USA, 2001. ISBN 9780419245506—CAT# RU29204.

[20] Marchand J.; Egege Samson; Maltais Y.; Beaudoin J.J. Theoretical Analysis of the Effect of Weak Sodium Sulfate Solutions on the Durability of Concrete. Cement and Concrete Composites 2002, 24, 317–329, 10.1016/S0958-9465(01)00083-X

[21] Gao R., Li Q.; Zhao S. Concrete deterioration mechanisms under combined sulphate attack and flexural loading. Journal of Materials in Civil Engineering 2013, 25, 39–44, 10.1061/(ASCE)MT.1943-5533.0000538

[22] Feng Ming; You-sheng Deng; Dong-qing Li. Mechanical and Durability Evaluation of Concrete with Sulphate Solution Corrosion. Advances in Materials Science and Engineering 2016, Article ID 6523878, 10.1155/2016/6523878

[23] Hong H. Assessment of reliability of aging reinforced concrete structures. J. Struct. Eng. ASCE 2000, 126, 1458–1465.

[24] Luping, T.; Andersen, A. Chloride ingress data from five years field exposed in a Swedish marine environment. In Proceedings of 2nd RILEM International Workshop on Testing and Modelling the Chloride Ingress into Concrete; C. Andrade and J. Kropp; Paris, France, 9–10 September 2000, 1–15.

[25] Bauer J.; Puła W. Some remarks on application of response surface method in reliability computations. W: Numerical models in geomechanics. NUMOG VII. Proceedings of the Seventh International Symposium on Numerical Models in Geomechanics, Graz, Austria, 1–3 September 1999 /Ed. by G. N. Pande, S. Pietruszczak, H.F. Schweiger. Rotterdam: A.A.Balkema, 1999, 221–228.

[26] Vessia G.; Kozubal J.; Puła W. High dimensional model representation for reliability analyses of complex rock-soil slope stability. Archives of Civil and Mechanical Engineering, 2017, 17, 954–963. ISSN: 1644-9665

[27] Lang A.; Potthoff J. Fast simulation of Gaussian Random Fields. Monte Carlo Methods and Applications 2011, 17, 195–214.

[28] Vanmarcke E. H. Probabilistic modeling of soil profiles. Journal of the Geotechnical Engineering Division, 1977, 103, 1227–1248.

[29] Vanmarcke E. H. Random fields: Analysis and synthesis; The MIT Press: Cambridge, USA, 1983. ISBN 0262720450.

[30] Ching J.; Phoon K.; Kao P. Mean and variance of mobilized shear strength for spatially variable soils under uniform stress states. Journal of Engineering Mechanics 2014, 140, 487–501, 10.1061/(ASCE)EM.1943-7889.0000667

[31] International Standard ISO 2394:2015. General principles on reliability for structures. International Standards Organisation, 2015

