# Supplementary material for: Probabilistic analysis of a concrete column in an aggressive soil environment
Source: PLoS One. 2019 Mar 7;14(3):e0212902. doi: 10.1371/journal.pone.0212902 (PMC6407909; doi:10.1371/journal.pone.0212902)
Supplement: S6 File — (PDF) [file pone.0212902.s006.pdf]

American Manuscript Editors

English Editing Certificate

This document certifies that the manuscript listed below was edited for proper English language, grammar, punctuation, and spelling by the expert staff at American Manuscript Editors.

Manuscript Title:

Probabilistic analysis of a concrete column in an aggressive soil environment

Authors:

Marek Wyjadlowski,
Janusz Kozubal,
Dmitri Steshenko

Certificate Verification Key:

115-049-611-129-654

Project Number:

57770

This certificate may be verified by emailing info@americanmanuscripteditors.com. Documents receiving this certificate should be prepared for publication. However, please note that the author has the ability to accept or reject our suggestions for changes and can make changes after the editing process is complete, all of which can adversely affect the quality of the text after the editing process.

AMERICAN MANUSCRIPT EDITORS
